# The dark side of mRNA translation and the translation machinery in glioblastoma

**DOI:** 10.3389/fcell.2023.1086964

**Published:** 2023-03-13

**Authors:** Angélica Montiel-Dávalos, Yeniffer Ayala, Greco Hernández

**Affiliations:** From the Translation and Cancer Laboratory, Unit of Biomedical Research on Cancer, National Institute of Cancer (Instituto Nacional de Cancerología, INCan), Mexico City, Mexico

**Keywords:** cancer, translation initiation, glioblastoma, ribosomal proteins (RP), eIF4E, translation machinery, TMZ (temozolomide)

## Abstract

Among the different types of cancer affecting the central nervous system (CNS), glioblastoma (GB) is classified by the World Health Organization (WHO) as the most common and aggressive CNS cancer in adults. GB incidence is more frequent among persons aged 45–55 years old. GB treatments are based on tumor resection, radiation, and chemotherapies. The current development of novel molecular biomarkers (MB) has led to a more accurate prediction of GB progression. Moreover, clinical, epidemiological, and experimental studies have established genetic variants consistently associated with the risk of suffering GB. However, despite the advances in these fields, the survival expectancy of GB patients is still shorter than 2 years. Thus, fundamental processes inducing tumor onset and progression remain to be elucidated. In recent years, mRNA translation has been in the spotlight, as its dysregulation is emerging as a key cause of GB. In particular, the initiation phase of translation is most involved in this process. Among the crucial events, the machinery performing this phase undergoes a reconfiguration under the hypoxic conditions in the tumor microenvironment. In addition, ribosomal proteins (RPs) have been reported to play translation-independent roles in GB development. This review focuses on the research elucidating the tight relationship between translation initiation, the translation machinery, and GB. We also summarize the state-of-the-art drugs targeting the translation machinery to improve patients’ survival. Overall, the recent advances in this field are shedding new light on the dark side of translation in GB.

## Introduction

Brain tumors include astrocytomas, oligodendrogliomas, and glioblastoma (GB) (Louis et al., 2021). The World Health Organization (WHO) determined that GB, a high-grade (WHO grade 4) tumor, is the most frequent and malignant nervous system tumor. Indeed, about half of the malignant brain tumors are GB. Incidence rates of GB are higher in men than women and Whites than in Blacks. GB occurs predominantly in the brain, being rather seldom in the brain stem, cerebellum, and spinal cord (Michaud, 2020; McKinnon et al., 2021; Quinones and Le, 2021; Thakur et al., 2022). Histologically, GB displays characteristics such as fast tumor growth, nucleus alterations, and changes in cell morphology and size (D’Alessio et al., 2019). In 2021, WHO classified GB using tumor grade, DNA methylome, and histopathologic, immunohistochemical, ultrastructural, and molecular diagnostic (Louis et al., 2021; Bale and Rosenblum, 2022).

WHO classifies diffuse gliomas according to histological grades 2 or 3 as highly metastatic tumors of the CNS. Diffuse gliomas show high relapse rates. Grade 4 GB is the most frequent and aggressive tumor among adults, with a 16–18 months of survival (Louis et al., 2021). GB treatment is mainly based on surgical resection and radio- and temozolomide (TMZ)-based chemotherapies. Most current treatment protocols include bevacizumab, nitrosoureas, checkpoint inhibitors for CAR T cells, and oncolytic viruses, among others (Altshuler et al., 2020; Quinones and Le, 2021; Rodríguez-Camacho et al., 2022). Unfortunately, the current therapies have not extended the 15-month survival outcome of GB patients.

Recently, large-scale genomic and epigenomic studies have discovered various genetic alterations underlying GB development. Well-studied GB-associated mutations include changes in the following genes: *cyclin-dependent kinase inhibitor 2B antisense RNA 1(CDKN2B-AS1)*, *isocitrate dehydrogenase 1* (*IDH*), *tensin phosphatase homolog deleted on chromosome 10 (PTEN)*, *loss of heterozygosity of chromosome 10* (*LOH 10q*), *epidermal growth factor receptor* (*EGFR*) amplification, *Mouse double minute 2* (*MDM2*), *telomerase reverse transcriptase* (*TERT*) promoter, *Fat1protocadherin* (*FAT1*), *alpha thalassemia/X-linked intellectual disability syndrome* (*ATRX*), *TP53* (encoding the tumor suppressor protein p53), and *death domain–associated protein* (*DAXX*) (Lee et al., 2018; Dahlin et al., 2019; Silantyev et al., 2019).

Some GB molecular biomarkers (MB), including mutations in isocitrate dehydrogenase (IDH 1 and 2), epidermal growth factor receptor (EGFR), and p53, among others, have been identified, which allow for predicting tumor progression and, ultimately, helping increase patient survival (Pierscianek et al., 2019; Birkó et al., 2020; Senhaji et al., 2022). High plasma levels of Insulin-like growth factor binding protein 2 (IGFBP-2) are also a prognosis marker in GB patients. Other MB focus on the methylation status of the *O6-methylguanine DNA methyltransferase* (*MGMT*) gene promoter. The *epidermal growth factor* (*EGFR*) gene may show splicing variants, mutations, rearrangements, and amplification in GB patients. Mutations in the telomerase *TERT* gene and its regulatory regions, and in the genes *Loss of Heterozygosity* (*LOH*) and *TP53*, also might be established as GB MB. Some of these MB help predict survival and response to TMZ therapies (Rao et al., 2018; Pierscianek et al., 2019; Birkó et al., 2020; Senhaji et al., 2022).

Despite the advances in the molecular biology of GB, fundamental processes inducing tumor onset and progression remain to be elucidated. Translation, i.e., decoding the genetic message of an mRNA into polypeptide by the ribosome and translation factors, is emerging as a significant cause of GB when defective. In this review, we focus on the relationship between GB and the initiation step of translation, the translation initiation factors (eIFs), the transduction cascades signaling to translation initiation, and ribosomal proteins (RPs). Moreover, we describe current advances in drugs targeting the translation process and its regulation as part of new strategies to treat GB.

## Dysregulation of translation initiation as a key cause of cancer

Translation plays a crucial role in the composition and quantity of the cellular proteome in all forms of life and diseases (Tian et al., 2004; Vogel et al., 2010; Schwanhausser et al., 2011; Hernández and Tettweiler, 2012; Wang et al., 2013). Thus, dysregulated translation is a cause of cancer development. Indeed, various translation factors are oncogenes, others are overexpressed in tumors, and some components of the translational machinery are mutated across different types of cancer. During carcinogenesis, tumor cells rapidly proliferate upon a significant increase in protein synthesis (Ali et al., 2017; Robichaud et al., 2018; Xu and Ruggero, 2020; Fabbri et al., 2021). Moreover, malfunctioning of the signal transduction cascades controlling translation may promote neoplastic phenotypes (Roux and Topisirovic, 2018; Proud, 2019).

### An overview of translation initiation

The cellular structures where mRNA translation takes place are the ribosomes, which are integrated by two subunits. Ribosomal subunits are macromolecular ribonucleoprotein complexes composed of ribosomal RNA (rRNA) and ribosomal proteins (RPs). In eukaryotes, the large (L) subunit 60S possesses three rRNA, 5S, 5.8S, 28S, and 47 proteins termed RPL. The small (S) 40S subunit contains an 18S rRNA and 33 proteins termed RPS. RPs primarily participate in ribosome assembly and protein synthesis (Weisser and Ban, 2019).

**FIGURE 1:**
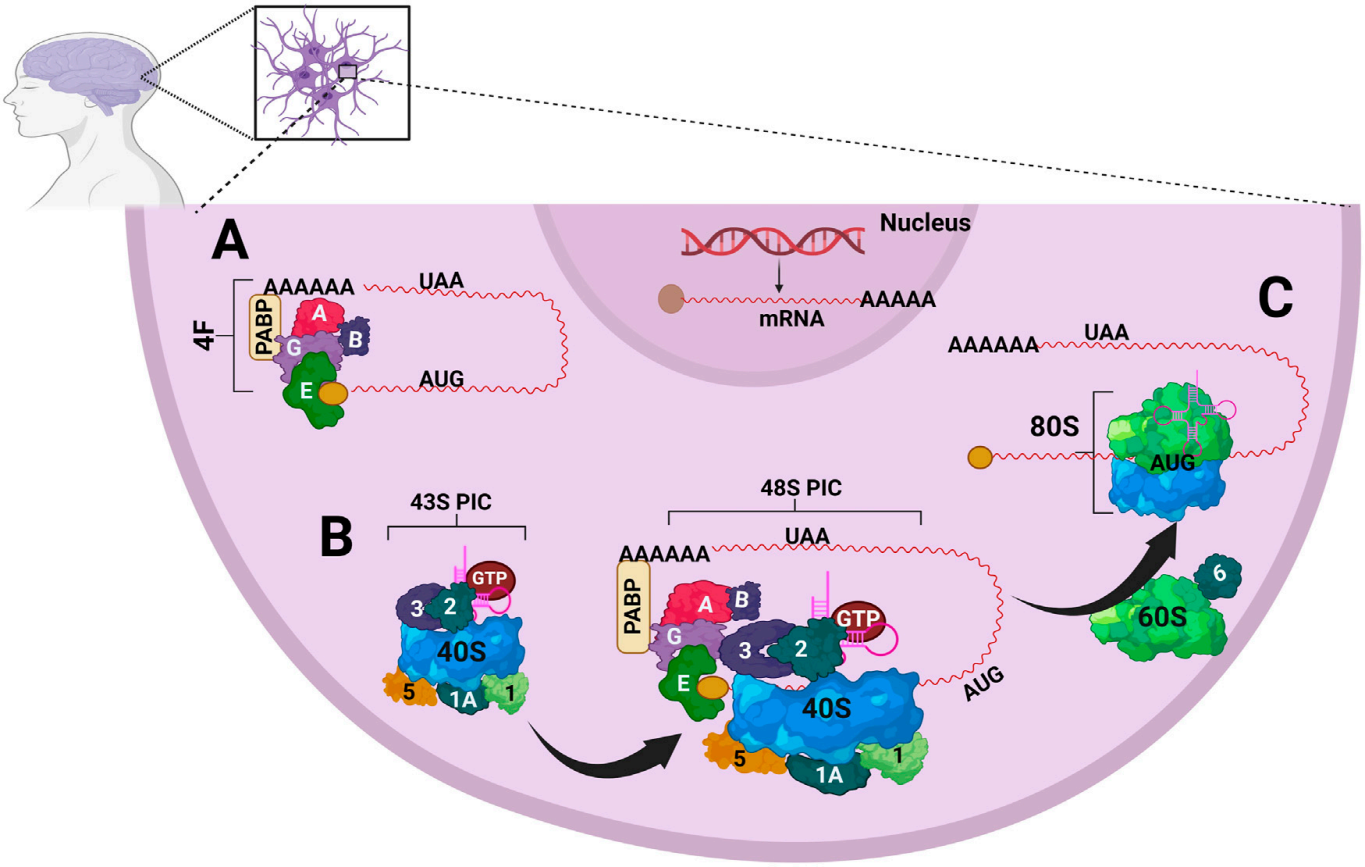
A summary of the translation initiation. eIFs are shown only with the respective *numbers*. **(A)** This process begins with forming the eIF4F complex (*4F*). **(B)**. A free 40S ribosomal subunit interacts with eIF1, eIF1A, eIF3, eIF5, and the TC (eIF2●Met-tRNAi^Met^●GTP), forming a *43S* pre-initiation complex (*PIC*). A 43S PIC is recruited to the 5′ end of the mRNA through the interaction of eIF3 and eIF4G bound to the ribosome resulting in the formation of a *48S PIC*. Poly(A) binding protein (*PABP*) binds to the poly(A) tail at the 3′ end of the mRNA and to eIF4G. These interactions circularize the mRNA. **(C)** GTP-eIF5B promotes eIF1 and eIF5B release, facilitating the association of a 60S subunit to the 48S PIC resulting in the assembly of an *80S* initiation complex.

The translation process is divided into phases initiation, elongation, termination, and ribosome recycling, and the whole process is mostly regulated at the initiation phase (Hershey et al., 2019). In eukaryotes, translation initiation consists of the recruitment of an mRNA to the ribosome and placement of the mRNA translation initiation site (TIS) in the peptidyl (P) site of the 40S ribosomal subunit to establish the mRNA reading frame (Hershey et al., 2019). During the initiation phase, two checkpoints represent the major targets of regulation. One of them is the recognition of the cap structure 7-methylguanosine (m^7^GpppN) at the 5′-end of the mRNA by the cap-binding protein eIF4E (Figure 1A). For this to occur, eIF4E makes up a complex with the scaffold protein eIF4G and the RNA helicase eIF4A, the so-called eIF4F complex. Next, the poly(A)-binding protein (PABP) binds to the poly(A) tail at the 3′-end of the mRNA and eIF4G, thus promoting mRNA circularization. Simultaneously, a free 40S ribosomal subunit interacts with eIF1, eIF1A, eIF3, eIF5, and the ternary complex (TC, consisting of eIF2 bound to an initiator Met-tRNA_i_^Met^ and GTP) to form a 43S pre-initiation complex (PIC) (Figure 1B). The 43S PIC is then recruited at the 5′-end of the mRNA through the interaction of ribosome-bound eIF3 and eIF4G. These interactions drive the formation of a 48S PIC (Figure 1B). Afterward, eIF4A unwinds stem-loop structures of 5′-untranslated region (UTR), allowing the 43S PIC to scan the 5′-UTR to reach a TIS (usually an AUG codon) (Hinnebusch, 2014; Lind and Åqvist, 2016; Pelletier and Sonenberg, 2019).

When a TIS is reached, eIF1 and eIF1A drive the faithful selection of a TIS. In this process, the initiator Met-tRNA_i_^Met^ is positioned in the ribosome’s peptidyl (P) site, making Watson-Crick base-pairing between the start codon AUG and the anticodon of the Met-tRNA_i_^Met^. As a result, scanning arrests and the Met-tRNA_i_^Met^ and eIF1A get tightly positioned within the P-site. Then, GTP-eIF5B promotes the release of eIF1 and eIF5B, facilitating the joining of a 60S subunit to the 48S PIC to form an 80S initiation complex, which is competent to start translation elongation (Figure 1C) (Hinnebusch, 2014; Lind and Åqvist, 2016; Pelletier and Sonenberg, 2019).

A second major point of regulation relies on the eIF2 activity. eIF2 is formed by *α*, *β*, and *γ* subunits. eIF2 activity is regulated by the reversible phosphorylation of eIF2α Ser51, being p-eIF2α (the phosphorylated form of eIF2α) an inhibitor of global protein synthesis. In addition, different kinases phosphorylate eIF2α: the heme-regulated inhibitor kinase (HRI) that is activated under heme deprivation or arsenite exposure; the double-stranded RNA protein kinase (PKR) that responds to viral infection; the general control non-derepressible 2 (GCN2) that is activated by uncharged tRNA and thus senses amino acid starvation; and the PKR-like endoplasmatic reticulum kinase (PERK) that is activated by unfolded proteins in endoplasmic reticulum (Wek, 2018).

### Signal transduction controlling translation initiation

**FIGURE 2:**
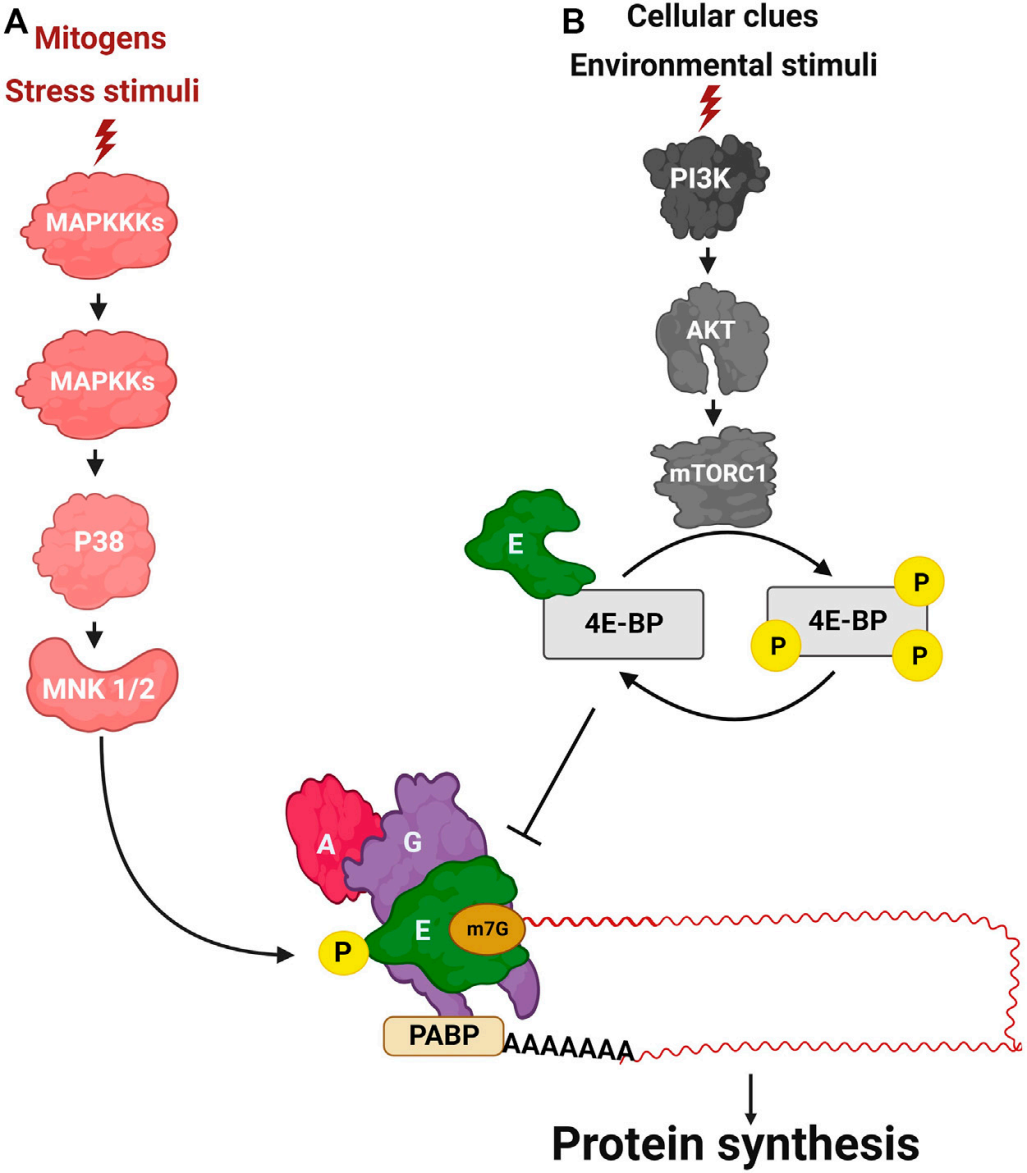
The transduction cascades signaling to translation initiation. **(A)** The MAPK pathway. **(B)** The PI3K/AKT/mTOR pathway.

Two major pathways signal to the initiation phase of translation, namely, the mitogen-activated protein kinases (MAPK) pathway and the phosphatidylinositol 3-kinase (PI3K)/protein kinase B (Akt)/mammalian target of rapamycin complex 1 (mTORC1) pathway (Figure 2A). MAPKs are activated by mitogens and stress stimuli, and are coupled to the translation machinery *via* the downstream phosphorylation of Mitogen-activated protein kinase-interacting kinase 1 and 2 (MNK1/2) that phosphorylate eIF4E (Roux and Topisirovic, 2018; Proud, 2019). On the other side, the mTORC1 pathway senses, integrates, and drives responses to stress, cellular energy status, nutrient availability, hormones, and mitogens to control cellular proliferation and survival (Figure 2B). In response to these cues, the mTOR cascade controls translation initiation *via* eIF4E-binding proteins 1 and 2 (4E-BP1/2) which bind to eIF4E depending on their phosphorylation status: whereas dephosphorylated 4E-BPs bind eIF4E, phosphorylated proteins dissociate from eIF4E. Interaction of 4E-BPs to eIF4E inhibits the formation of the eIF4E/eIF4G complex, thereby repressing cap-dependent translation (Fonseca et al., 2016; Roux and Topisirovic, 2018).

## Translation and GB in the spotlight

Recently, malfunctioning of the translation process has emerged as very relevant in GB development. Indeed, changes in translation efficiency represent the primary mechanism governing cellular responses to changes in oxygen concentration in GB cells (Ho et al., 2016). This is crucial because the microenvironment within tumors becomes highly hypoxic, a condition that triggers a switch from a normoxia translation machinery to an alternative hypoxia machinery (Uniacke et al., 2012; Uniacke et al., 2014; Ho et al., 2016; Timpano and Uniacke, 2016). We describe the involvement of the translational machinery in GB’s onset and progression in the following, i.e., the translation initiation factors, the transduction cascades signaling to eIF4E, and the RPs. We next review the reprogramming that the initiation of translation undergoes under anaerobic metabolism. Finally, we summarize the advances in pharmacological research targeting translation initiation aiming to alleviate this malady.

### eIFs of the 43S PIC

**FIGURE 3:**
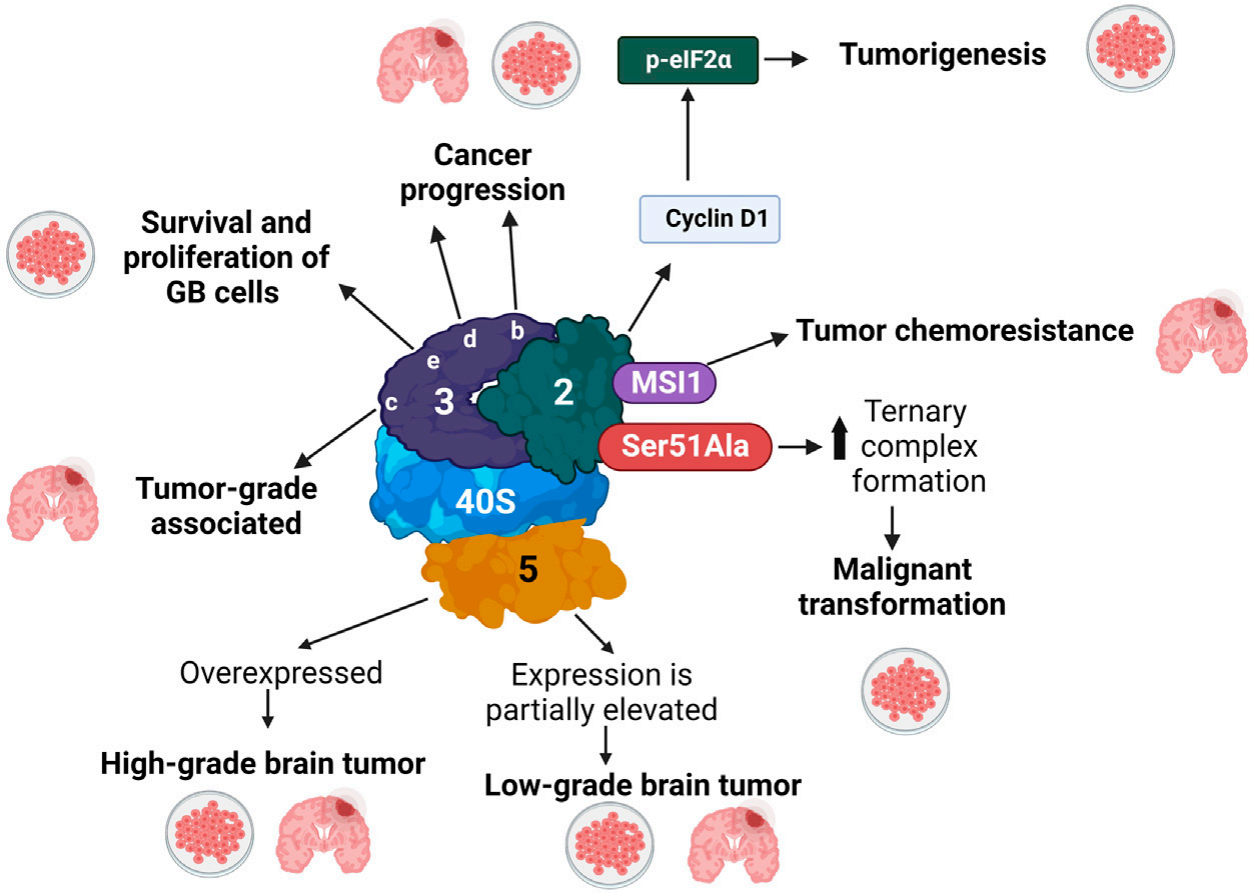
eIFs components of the 43S PIC involved in GB progression. eIFs are shown only with the respective *numbers* in complex with the *40S* ribosomal subunit. *Thick arrows* indicate overexpression (upward) or downregulation (downward). *Thin arrows* indicate what GB process eIFs are involved in. The different phenomena observed in patients and in cultured cells, are indicated with a *Petri dish* and a *brain*, respectively.

**FIGURE 4:**
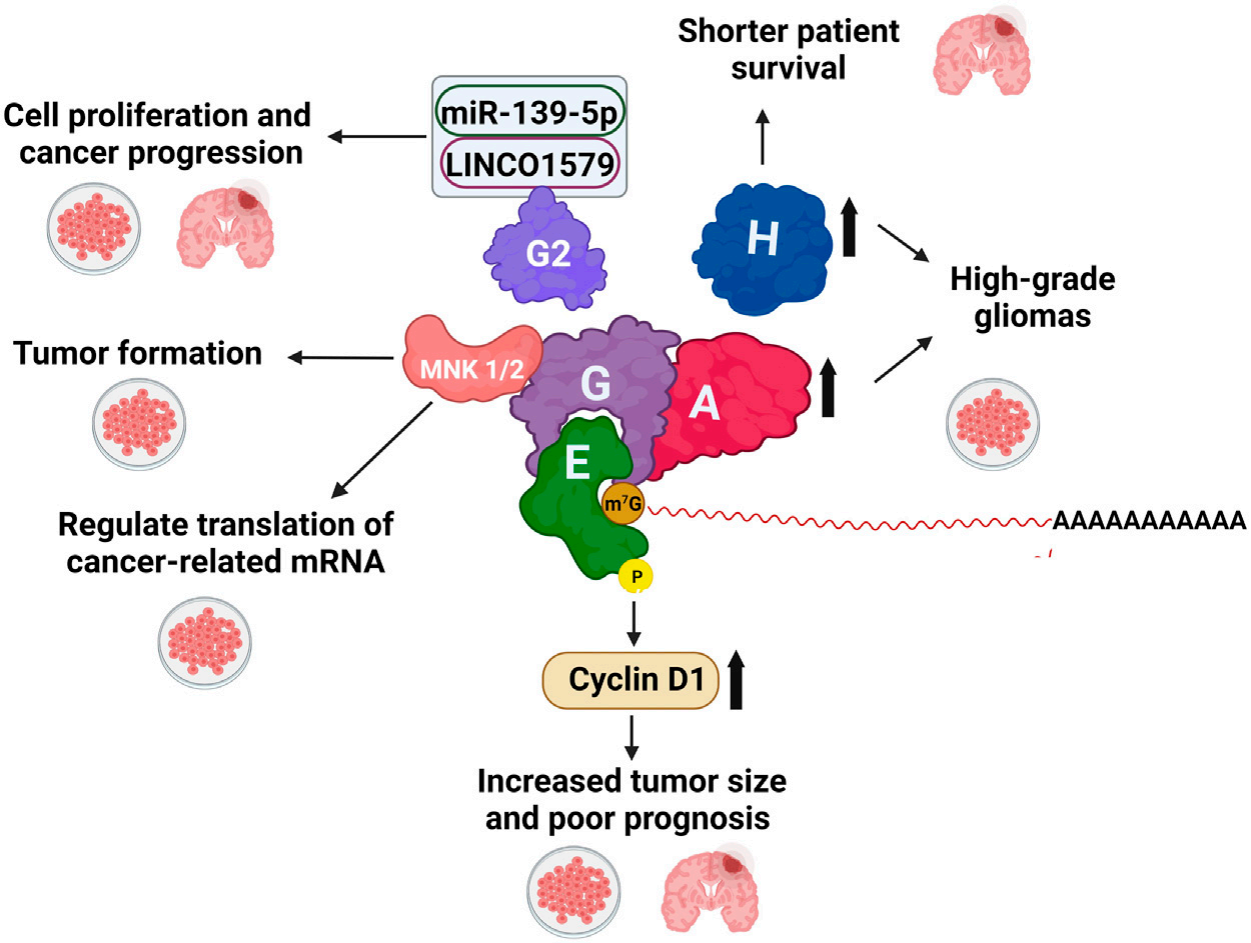
The eIF4F complex and the MNK1/2-eIF4E axis are involved in GB progression. eIFs are shown only with the respective *numbers*. *Thick arrows* indicate overexpression (*upward*) or downregulation (*downward*). *Thin arrows* indicate what GB process eIFs are involved in. The different phenomena observed in patients and in cultured cells, are indicated with a *Petri dish* and a *brain*, respectively.

We describe the relevance of eIFs in GB biology. First, we give an account of the eIFs integrating the 43S PIC (Figure 3), followed by the components of the eIF4F complex (Figure 4).

#### eIF2

eIF2 has been associated with cancer (Figure 3). Early studies established that the ectopic expression of a non-phosphorylatable eIF2α mutant Ser51Ala promoted the free activity of the ternary complex, driving continuous, unregulated translation initiation, which may lead to malignant transformation of cells. Accordingly, a dominant-negative PKR mutant caused oncogenic phenotypes in immortalized cells (Donze et al., 1995). In patients with different types of cancer, including sarcomas, various lymphomas, melanomas, gastric tumors, thyroid carcinomas, lung tumors, and malignant brain tumors, eIF2α levels are upregulated, which might promote an increase in the rate of protein synthesis (Aktas and Chen, 2014). Another study reported that the interaction of eIF2 with Musashi1 (MSI1), an RNA-binding oncoprotein that controls the balance between self-renewal and differentiation during neurogenesis (Velasco et al., 2019), promoted tumor chemoresistance in GB cells (Koromilas, 2015). MS1 has been strongly related to different brain tumors, including medulloblastoma and GB (Velasco et al., 2019). Tejada et al. (2009) studied the expression, subcellular location, phosphorylation state, expression of cyclin D1 and its correlation with eIF2α in different brain tumors, including astrocytomas, meningiomas, ganglioglioma, oligodendroglial tumors, and neurinomas. They observed that the expression and phosphorylation of eIF2α in these tumors are involved in the synthesis of cyclin D1, most probably downregulating its expression.

#### eIF3

Human eIF3 is composed of 13 subunits (a-m). With a molecular weight of ∼800 kDa, it is the largest and most complex initiation factor (des Georges et al., 2015; Eliseev et al., 2018). eIF3 promotes the assembly of the 43S PIC with the mRNA-bound eIF4F, scanning, and TIS recognition (Jivotovskaya et al., 2006; Stanciu et al., 2021). eIF3 is also involved in translation termination, ribosome recycling, and stop codon read-through scanning (Beznosková et al., 2013; Beznosková et al., 2015; Hellen, 2018).

The mRNAs of all eIF3 subunits, except subunit k, are significantly overexpressed in GB (Digregorio et al., 2019), and the levels of eIF3 proteins are increased in high-grade gliomas (Krassnig et al., 2021). These observations are important because the following eIF3 subunits have been involved in GB progression (Figure 3). eIF3b is strongly expressed in GB patients and the GB cell lines U251, U373, U-87 MG, and A172 (Liang et al., 2012), and a reduction of eIF3b levels in U-87 MG cells decreased cell proliferation, arrests the cell cycle, and triggered apoptosis (Liang et al., 2012). eIF3c was found highly expressed in glioma samples compared to different non-cancer brain tissues, and eIF3c levels were associated with tumor grades. Accordingly, the siRNA-induced reduction of eIF3c levels in U-87 MG cells arrested cell proliferation and colony formation and triggered cell cycle arrest (Hao et al., 2015). eIF3d was also discovered to be involved in the progression of GB (Lee et al., 2016; Chai and Xie, 2019; Bertorello et al., 2020). An immunohistochemical study showed that eIF3d is overexpressed in grade-1 and 2 GB brain tumors and U251 and U-87 MG glioma cells (Ren et al., 2015), and when eIF3d was knocked out, the cell proliferation and colony formation decreased. Due to its participation in regulating the proliferation and migration of GB cells, eIF3d could be a therapeutic candidate for GB (Ren et al., 2015). Finally, eIF3e was found essential for the survival and proliferation of GB cells (Sesen et al., 2014).

#### eIF5

The GTPase eIF5 plays a key role in 48S PIC assembly (Pestova et al., 2000; Pisareva and Pisarev, 2014). eIF5 has been linked to GB (Figure 3), although its role in carcinogenesis has not been elucidated. *In vitro* studies, the GB cell lines G55T2 and U-87-MG also showed eIF5 overexpression compared to primary normal human astrocytes (NHA) (Preukschas et al., 2012). eIF5 was overexpressed in high-grade GB patients compared to low-grade tumors and non-neoplastic controls, while eIF5 expression is partially elevated in low-grade samples (Krassnig et al., 2021). In addition, GB patients with high eIF5 expression had a poor prognosis for survival (McLendon et al., 2008).

### eIFs of the eIF4F complex

#### eIF4E

Among the components of the translational machinery, eIF4E is the most studied protein in patients and in both normal and cancer cells. eIF4E is overexpressed in a wide variety of tumors and is a marker of poor prognosis (Ali et al., 2017; Robichaud et al., 2018). Indeed, the *eIF4E* gene is a proto-oncogene (De Benedetti and Rhoads, 1990; Lazaris-Karatzas et al., 1990) whose overexpression drives an increase in tumorigenesis, invasion, and metastases in mouse models. Accordingly, the impairment of eIF4E activity by high levels of 4E-BPs or the use of antisense RNAs decreased the carcinogenic phenotypes in different cells and animal models and increased cell sensitivity to chemotherapeutic drugs (Ali et al., 2017; Robichaud et al., 2018; Xu and Ruggero, 2020; Fabbri et al., 2021). Moreover, phosphorylated eIF4E (p-eIF4E) is often upregulated in different cancer types, and phosphorylation of eIF4E is involved in essential processes in cancer biology, including cell transformation, proliferation, apoptosis, metastasis, and angiogenesis (Yang et al., 2020).

Overall, eIF4E oncogenic activity is primarily due to its capacity to translate a specific subset of mRNAs involved in cancer progression at a higher rate. mRNAs containing short (100–200 nucleotides) and unstructured 5′-UTRs facilitate the scanning process and are considered “strong” because they are easily translated in low concentrations of the eIF4F complex (formed by eIF4E, eIF4G, and eIF4A). In contrast, “weak” (also termed “eIF4E-sensitive”) mRNAs have long, structured, and GC-rich 5′-UTRs and are feebly translated (Koromilas et al., 1992; Pesole et al., 2001; De Benedetti and Graff, 2004; Pickering and Willis, 2005). Restraining eIF4E availability by the action of 4E-BP led to a preferential inhibition of translation of weak transcripts with an impact on cancer progression (De Benedetti and Graff, 2004; Hsieh et al., 2012; Musa et al., 2016). Thus, eIF4E upregulation promoted to overexpression of a subset of weak mRNAs involved in cell cycle progression and proliferation, tumor growth, apoptosis inhibition, metastasis, and angiogenesis, such as nuclear factor of activated cells (NFAT), c-Myc, Ornithine decarboxylase (ODC), fibroblast growth factor-2 (FGF2), Vascular endothelial growth factor (VEGF), Cyclin D1 (Kevil et al., 1995; Kevil et al., 1996; Nathan et al., 1997; Graff and Zimmer, 2003; Wendel et al., 2004; Mamane et al., 2007; Wendel et al., 2007). Weak mRNAs are also highly dependent on the RNA helicase eIF4A. Svitkin et al. showed that the level of translation inhibition by the action of eIF4A mutants was proportional to the degree of secondary structure present in the 5′-UTR mRNA (Svitkin et al., 2001).

The involvement of eIF4E and phosphorylated eIF4E in Ser209 (p-eIF4E) in brain carcinogenesis of different tumor types is well established (Table 1). In GB (Figure 4), Western blot and immunohistochemistry analyses showed that eIF4E and p-eIF4E were overexpressed in proliferative endothelial and vascular GB cells, as well as in lower-grade glioma, which is in most cases associated with adverse patient prognosis (Gu et al., 2005; Yang et al., 2007; Tejada et al., 2009; Martínez-Sáez et al., 2016; Liang et al., 2022). Moreover, overexpression of Cyclin D1 strongly correlated with p-eIF4E levels in GB (Tejada 2009). Accordingly, knocking down eIF4E led to a decrease in GB U251 cells’ capacity to proliferate and migrate and an increase in apoptosis (Liang et al., 2022). Thus, the potential value of p-eIF4E as a diagnostic tool in GB biopsies has also been proposed (Martínez-Sáez et al., 2016).

**TABLE 1:** eIF4E involvement in the development of brain tumors.

Model evaluated	Phenotype observed	References
High-grade astrocytic tumors	Prominent staining for eIF4E in the cytoplasm (but not the nucleus) of pyramidal neurons. In contrast, in neuroglia cells no overexpression of this protein was observed. This might be due to the high rates of metabolism that pyramidal cells show.	Gu et al. (2005)
Astrocytoma and reactive gliosis	Elevated levels of p-MNK1 and p-eIF4E correlate with tumor grade and with an increase in Cyclin D1 expression, which is associated with tumor recurrence, increased tumor size, and poor prognosis.	Bredel et al. (2005), Martínez-Sáez et al. (2016), Fan et al. (2017b)
Astrocytoma	eIF4E mRNA and protein expression increases with the increase of tumor grade.	Krassnig et al. (2021)
Oligodendroglial tumors	eIF4E is localized to the nucleus.	Tejada et al. (2009)
Meningiomas and oligodendroglial tumors	Elevated levels of eIF4E.	Tejada et al. (2009)
Astrocytic tumors	Significant correlation between p-eIF4E and Cyclin D1 expression.	Tejada et al. (2009)
Glioma initiating-cells	eIF4E promotes translation of the transcription factor SOX2, a key protein for maintaining self-renewal, or pluripotency, of embryonic stem cells, and neural stem cells.	Ge et al. (2010)

#### eIF4G

eIF4G is a scaffold protein that binds to and coordinates the activity of different factors for the assembly of the 48S PIC. During this process, eIF4G interacts with eIF4E and eIF4A forming the eIF4F complex for mRNA recruitment (Tahara et al., 1981; Pestova et al., 1996; Marcotrigiano et al., 2001). The human genome encodes three eIF4G isoforms, namely, eIF4G (Yan et al., 1992), eIF4G2 (also called NAT1, 97, and DAP-5) (Imataka et al., 1997; Levy-Strumpf et al., 1997; Shaughnessy et al., 1997; Yamanaka et al., 1997), and eIF4G3 (also called eIF4G II (Gradi et al., 1998).

eIF4G activity has been strongly related to breast, lung, and head and neck cancers (Wagner et al., 2014). Further metadata studies from the brain datasets REMBRANDT (Gusev et al., 2018) and the Cancer Genome Atlas (TCGA) (Verhaak et al., 2010) revealed that *eIF4G* and *eIF4G2*, but not *eIF4G3* mRNAs are highly overexpressed in different types of GB (Digregorio et al., 2019). However, the relevance of this observation in cancer is unknown. Chai et al. reported that long intergenic non-protein coding RNA 1579 (LINC01579) is overexpressed in GB tumors and promotes cell proliferation in the GB cell lines U251, and U87. Furthermore, LINCO1579 interaction with miR-139-5p upregulates eIF4G2 and promotes cell proliferation and cancer progression in GB cells (Chai and Xie, 2019). Accordingly, LINC01579 KO in cells inhibits proliferation and may lead to programmed cell death (Figure 4). As described below, eIF4G3 plays a crucial role in GB.

#### eIF4A and eIF4H

eIF4A is a highly conserved DEAD-box ATP-dependent RNA helicase key for scanning during mRNA translation (Grifo et al., 1984; Ray et al., 1985). In mammalian cells, eIF4H stimulates ATP binding to eIF4A, ATP-dependent RNA-binding, RNA-dependent ATPase, and helicase activity of eIF4A (Richter-Cook et al., 1998).

Dysregulation of eIF4A activity promotes cancer phenotypes such as cell invasion, proliferation, migration, and epithelial-mesenchymal transition in different neoplasia, including lung adenocarcinoma (Wu et al., 2022), breast (Modelska et al., 2015), colorectal (Li et al., 2017), and gastric cancers (Gao et al., 2020) in which eIF4A gene is overexpressed and eIF4A levels associated to poor clinical outcomes. In high-grade gliomas, eIF4A and eIF4H are significantly increased, and their expression level is related to the degree of tumor progression (Figure 4). Moreover, in GB, high eIF4H gene expression correlates with shorter patient survival (Digregorio et al., 2019; Krassnig et al., 2021).

### Ribosomal proteins

Early studies in Zebrafish reported that the loss of some ribosomal proteins (RPs) increases cancer risk (Amsterdam et al., 2004), suggesting a tumor suppressor ability. More recently, RPs have been reported to have both pro- and anti-tumoral activities. However, their specific role in translation (if any) remains unknown.

RPL5 is a significant candidate tumor suppressor in GB and other types of cancer, including aggressive, chronic lymphocytic leukemia, melanoma, and breast cancer. *RPL5* is heterozygously mutated in about 2.6% of GB tumors (Fancello et al., 2017). *RPL5* is also heterozygously deleted in about 11% of GB tumors, and patients expressing low RPL5 have poorer prognoses than patients expressing high RPL5 levels (Fancello et al., 2017). Both types of injuries could impact the RPL5/RPL11/5S rRNA complex assembly that binds to MDM2 and blocks MDM2-mediated ubiquitination of p53, leading to p53 stabilization in GB tumors with anti-tumoral effects (Orsolic et al., 2020).

RPS27, also termed metallopanstimulin-1 (MPS1), is an oncogenic protein in different cancers involved in cell invasion and migration through integrin β4 (ITGB4) activity, which is a downstream target of RPS27 (Yang et al., 2012), and MDM2-p53 signaling (Xiong et al., 2011). RPS27 is overexpressed in GB and gliomas of grade II/III, glioma stem-like cells, and macrophages associated with the tumor tissue. Moreover, RPS27 is released from tumors and can be measured in the patient’s serum. Therefore, this could be useful as a tumor marker for early detection (Feldheim et al., 2020a; Feldheim et al., 2020b).

**FIGURE 5:**
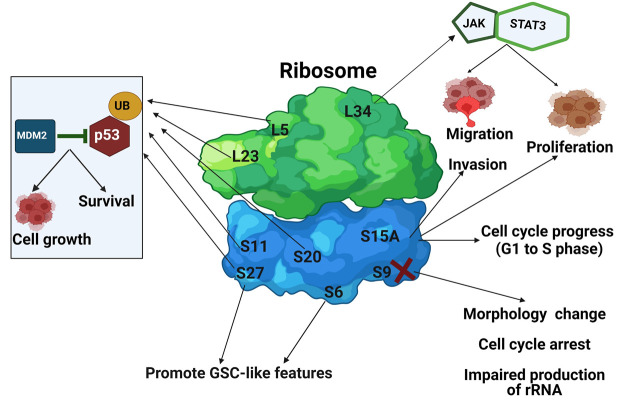
The involvement of RPs in GB. The 60S and 40S ribosomal subunits are depicted in *green* and *blue*, respectively. RPs are shown as *L* or *S* and their respective number. *Arrows* indicate what GB process RPs are involved in.

Other RPs are commonly overexpressed in GB tumors, including RPS11, RPS20, RPL5, and RPS27 (Yong et al., 2015; Fancello et al., 2017; Feldheim et al., 2020a; Vastrad et al., 2020). The involvement of different RP in the GB biology is summarized in Table 2 and Figure 5.

**TABLE 2:** Ribosomal proteins involved in GB progression.

Ribosomal protein	Function	References
RPS11, RPS20, RPL23, RPL5, and RPS27	Inhibit MDM2-mediated p53 ubiquitination and regulate cell growth and survival.	Xiong et al. (2011), Yong et al. (2015), Fancello et al. (2017), Orsolic et al. (2020)
RPS6, phosphorylated RPS6 (Ser235/236) and, RPS27	Promote the formation of glioblastoma stem cell-like characters.	Feldheim et al. (2020a), Shirakawa et al. (2020), Shirakawa et al. (2021)
RPS9	Depletion of RPS9 induces change in morphology, p53 dependent cell cycle arrest, and impaired production of 18S ribosomal RNA.	Lindström and Nistér (2010)
RPL34	Triggers proliferation, migration, and invasion through JAK/STAT3 signaling pathway.	Ji et al. (2019)
RPS15A	Involved in cell cycle progress from G1 to S phase, survival, proliferation, migration, and apoptosis *via* the mitochondrial pathway.	Yao et al. (2016), Zhang et al. (2016)

### Acquisition of GB stem cell features

Non-tumor stem cells renew themselves, giving rise to new cells, such as neurons, astrocytes and oligodendrocytes, and maintaining homeostasis throughout the organism. In contrast, GB stem cells (GSC) lack control of cell proliferation and differentiation, causing an uncontrolled response. That is why GSC have been attributed the role of the high number of recurrences of CNS tumors (Prager et al., 2020). Despite the existence of several therapeutic strategies, the prognosis for GB patients has not improved over the past decades due to chemoresistance and high relapse after surgery, a phenomenon possibly due to the involvement of GSC (Prager et al., 2020). Relapses are partially due to the heterogeneous presence of GSC and glia-like cancer stem cells that express stemness markers (Bhaduri et al., 2020). GSCs retain their potential for self-renewal, resistance to chemo and radiotherapies, high capacity of DNA repair, and increased mitochondrial reserve (Bao et al., 2006; Vlashi et al., 2011; Shirakawa et al., 2020; Tang et al., 2021). Overexpression of RPs in GSCs is associated with poor prognostic, and RPL11, and RPS20 overexpression has been proposed as a marker of poor prognostic in newly diagnosed and primary GB tumors. RPL11 high expression also is positively associated with unmethylated O6-methylguanine-DNA methyltransferase (MGMT) and wild-type IDH (Yong et al., 2015).

*In vitro* experiments revealed the role of RPS6 in inducing stem cell-like features in GB. pRPS6 co-localized with the expression of Nestin and CD34-positive in the perinecrotic and perivascular GSCs niches (Shirakawa et al., 2021). Transfection of U251MG and U-87 MG cells with RPS6-specific siRNA (siRPS6) significantly decreased neurosphere formation and the average size of spheres, along with a substantial reduction of Nestin and SOX2 expression (Shirakawa et al., 2020). Furthermore, RPS6 phosphorylation on Ser235-Ser236 in glioma tissues was observed in high-grade gliomas. Moreover, RPS6 phosphorylation on residues 240–244 correlates with mTOR phosphorylation, which is associated with poor prognosis in patients with GB (Machado et al., 2018; Shirakawa et al., 2021). Again, a direct role of RPs in translation is not clear.

## Shedding skin under hypoxia

eIF4E2, also known as eIF4E-homologous protein (4E-HP), was discovered in a human brain library (Rom et al., 1998). eIF4E2 shares 30% identity with eIF4E and possesses two Trp ➜ Tyr changes in the residues binding to the mRNA 5′ cap structure. Thus, eIF4E2 binds the cap at a 100-fold lower affinity than eIF4E (Zuberek et al., 2007). Mammalian eIF4E2 is 5–10 times less abundant than eIF4E (Rom et al., 1998), and depending on the interacting partners for integrating different protein complexes, eIF4E2 either activates or inhibits translation initiation (Morita et al., 2012; Timpano and Uniacke, 2016; Jeong et al., 2019). eIF4E2 does not bind eIF4G and associates with 4E-BP with low affinity (Joshi et al., 2004). Moreover, eIF4E2 does not possess the eIF4E phosphorylatable Ser209, which suggests that it is not regulated by the MAPK signaling pathways.

eIF4E2 is strongly related to cancer. In 2003, an eight-gene signature that contains *EIF4E2* associated with metastasis and poor clinical outcome was discovered in solid primary tumors from prostate, lung, breast, colorectal uterus, ovary, and medulloblastomas (Ramaswamy et al., 2003). Later on, *EIF4E2* was found to be significantly overexpressed in metastatic lung carcinoma (Sato et al., 2011).

Recently, eIF4E2 was discovered to promote translation in GB U87MG cells during hypoxia (Uniacke et al., 2012; Ho et al., 2016; Timpano and Uniacke, 2016), a physiological feature thriving in the microenvironment of solid tumors. Indeed, forming a hypoxic core in GB tumors necessitates eIF4E2-directed translation (Uniacke et al., 2014). Accordingly, REMBRANDT and ATCG data showed that *eIF4E2* mRNA levels are highly overexpressed in GB, oligodendroglioma, astrocytoma, mesenchymal, neural, and proneural cells (Digregorio et al., 2019). Under this condition, cells depleted for eIF4E2 from different types of cancer, including the GB line U-87 MG, showed impaired proliferation and increased apoptosis (Uniacke et al., 2014).

**FIGURE 6:**
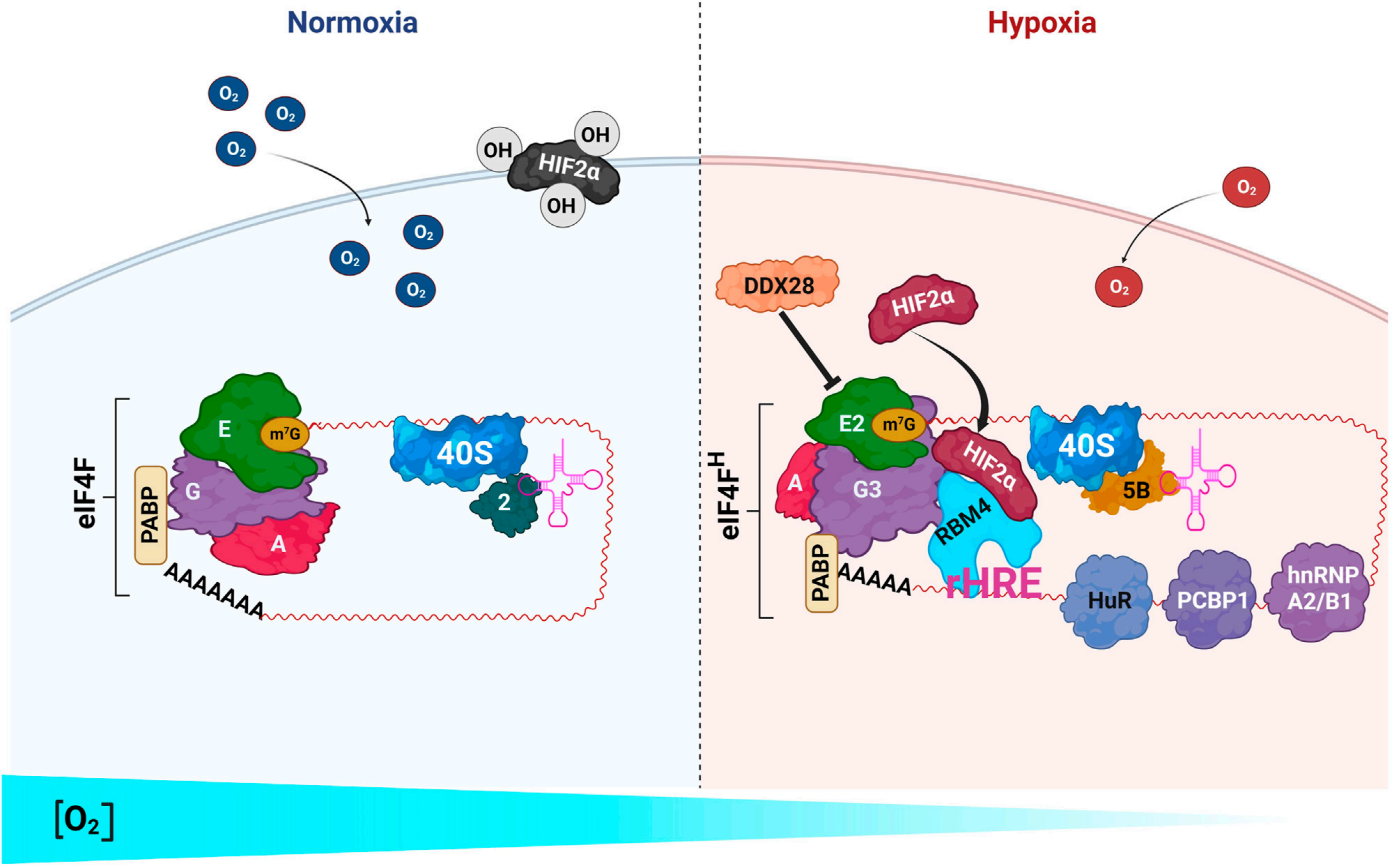
Two different machineries drive mRNA translation initiation under normoxia and hypoxia in GB. Only the relevant proteins are depicted. ***Left)*** A canonical *eIF4F* complex, formed by eIF4E (*E*), eIF4G (*G*), and eIF4A (*A*), drives mRNA translation initiation under normoxic conditions. ***Right)*** The switch to a high hypoxia condition leads to the assembly of a “hypoxic eIF4F” (*eIF4F* ^*H*^, formed by *eIF4E2*, *eIF4G3*, and *eIF4A*) that replaces the canonical eIF4F to drive the translation of the mRNAs possessing a hypoxia response element (*rHRE*). eIF4F^H^ also makes a complex with HIF2α and RBM4. During hypoxia, eIF5B, instead of eIF2, delivers tRNAs to the translating ribosome. Other RNA-binding proteins, such as HuR, PCBP1, and hnRNP A2/B1 bind the mRNAs being translated. The RNA helicase DDX28 represses HIF2α and eIF4E2 activities during eIF4E2-driven translation (Evagelou et al., 2020).

Strikingly, it was discovered in U-87 MG cells that the change from normal oxygen concentration to a highly anaerobic metabolism promoted a reconfiguration in the factors and RNA-binding protein (RBP) complexes that initiate mRNA translation. Upon a significant decrease in oxygen tension, cells repressed eIF4E and switched to an alternative cap-dependent translation mediated by eIF4E2 (Figure 6). In this process, the hypoxia-inducible factor (HIF)-2α interacted with RNA-binding motif 4 (RBM4) and also with eIF4E2 but not eIF4E. eIF4E2 further interacted with the eIF4G paralog eIF4G3 and the helicase eIF4A to form an active hypoxic eIF4F complex termed eIF4F^H^ (eIF4F hypoxia) that selectively promoted the translation of hypoxia-specific mRNAs, many of which encode proteins with roles in tumor progression such as in proliferation, survival, invasion, angiogenesis, and the evasion of apoptosis (Uniacke et al., 2012; Ho et al., 2016; Kelly et al., 2017; Ho et al., 2021). Accordingly, the inactivation of eIF4F^H^ significantly reduced tumor growth in a mice model (Uniacke et al., 2012). Hypoxia also caused the substitution of eIF2 by eIF5B for initiator methionine-tRNA delivery during translation initiation (Ho et al., 2018). Finally, the switch to an anaerobic metabolism promoted the formation of a network of hypoxia-sensitive RNA-binding proteins, including HuR, PCBP1, RBM4, and hnRNP A2/B1, that make a new complex with mRNAs for translation activation (Ho et al., 2020). The existence of two mRNA cap-binding complexes, namely, eIF4F and eIF4F^H^, makes possible the translation under a broad spectrum of oxygen concentrations, and both complexes can simultaneously be active within a stretch of that range (Uniacke et al., 2012; Ho et al., 2016; Timpano and Uniacke, 2016). Like serpents, under high hypoxia, mRNAs shed their skin.

## New drugs targeting translation in GB

**FIGURE 7:**
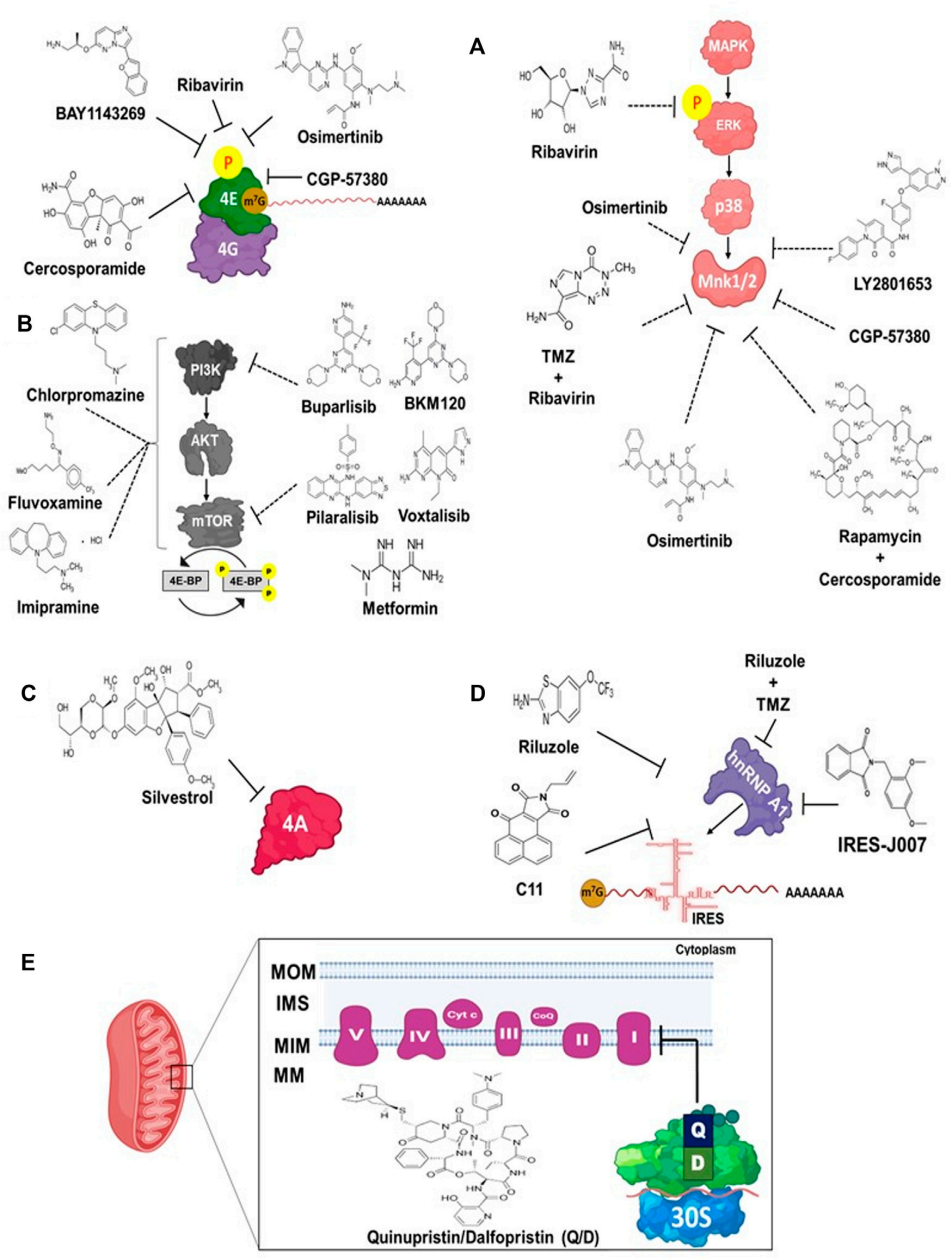
Chemical compounds targeting translation in GB. **(A)** Drugs targeting the eIF4E/eIF4G complex (***Left***) and the MAPK/ERK/p38/Mnk1 pathway (***Right***). **(B)** Drugs targeting the PI3K/AKT/mTOR pathway. **(C)** *Silvestrol* inhibits the eIF4A (*4A*) activity. **(D)** Compounds blocking the interaction between the hnRNP A1 protein with the Cyclin D1 and c-Myc IRES, thereby inhibiting IRES-dependent mRNA translation. **(E)** *Quinupristin* and *Dalfopristin* (*Q/D*) act synergistically to inhibit mitochondrial protein synthesis. These drugs bind to the mitoribosomal large subunit (*green*). *MOM*, mitochondrial outer membrane; *IMS*, Inter membrane space; *MIM*, Mitochondrial inner membrane; *MM*, mitochondrial matrix. *Solid lines* indicate a direct effect of the inhibitors on the translation machinery. *Dashed lines* indicate that the effect is not direct on the translation apparatus, but rather to the signaling cascades.

The current GB standard of care consists of surgical tumor resection followed by radiation and or TMZ chemotherapies (Singh et al., 2021). The effectivity of TMZ is highly dependent on the *O (6)-methylguanine-DNA methyltransferase* (*MGMT*) promoter methylation status, i.e., the highest the methylation of the promoter, the more effective TMZ against tumors is. MGMT is a DNA repair enzyme that fixes up damaged guanine nucleotides by transferring a methyl group at the O6 site of guanine to cysteine residues, thus reducing gene mutation and tumorigenesis. Therefore, patients with high methylation in CpG island of the *MGMT* promoter have better prognoses (Arora and Somasundaram, 2019; Yu et al., 2020). However, only about 50% of GB tumors show methylated *MGMT* promoter, and consequently, there is a high recurrence in patients with GB (Hegi et al., 2005). Therefore, therapies with new drugs are currently being studied. Among them, drugs directly targeting the translation machinery (eIF4E, eIF4A, IRES, or the mitoribosme) and indirectly targeting translation (MAPK or mTOR cascades) are up-and-coming (Figure 7).

### Targeting eIF4E and the MAPKs cascade

In response to mitogens and stress stimuli, extracellular-signal-regulated kinase (ERK) or p38 mitogen-activated protein (MAP) kinase activate downstream MNK1/2, which in turn phosphorylate eIF4E on Ser209 (Fukunaga and Hunter, 1997; Waskiewicz et al., 1997; Scheper et al., 2001). MNK1/2 interact with the carboxy-terminal of eIF4G in mammalian cells to directly phosphorylate eIF4E (Pyronnet et al., 1999). Due to the relevant role of eIF4E and p-eIF4E in cancer progression, dysregulation of the MNK1/2-eIF4E axis is linked to cancer in a wide spectrum of tissues (Yang et al., 2020; Xu et al., 2022). In GB, the MNK1-eIF4E axis regulates the translation of cancer-related mRNAs such as SMAD2, a key component of the TGF-β signaling pathway (Grzmil et al., 2011), and MNK1/2 are overexpressed in primary GB and glioma cells (Bredel et al., 2005; Grzmil et al., 2011; Xu et al., 2021). In addition, in GB U87MG cells, MNK1 knockdown resulted in a significant reduction in tumor formation when injected in a GB mice model (Ueda and al., 2010).

Several drugs target eIF4E and or the MAPK cascade to treat cancer (Figure 7A). Ribavirin is an antiviral drug that displays potent anti-cancer activity in breast, leukemia, and lung cancers (Pettersson et al., 2011; Urtishak et al., 2019; Tan et al., 2020). Previously, it was thought that these anti-cancer features were due to the ability to inhibit eIF4E activity through a structural similarity of ribavirin with the mRNA cap structure (Kentsis et al., 2004). Then, Westman et al. (2005) demonstrated that ribavirin does not compete with eIF4E for binding to the mRNA cap and impairs translation *in vitro*. Other experiments showed that ribavirin decreased eIF4E activity in glioma and GSCs by decreasing ERK and eIF4E Ser209 phosphorylation (Figure 7A) (Volpin et al., 2017). Although the mechanism underlying the antitumor effect of ribavirin on malignant gliomas cells remains unclear, this evidence suggests that ribavirin is involved in regulating the phosphorylation status of the ERK/MNK1/eIF4E signaling pathway (Shi et al., 2015; Volpin et al., 2017). More recent agents to treat GB include BAY1143269, a novel angiogenesis inhibitor with a potent anti-cancer activity (Wan et al., 2022). BAY1143269 inhibits eIF4E-mediated expression of oncogenic proteins, including those involved in the cell cycle and the epithelial-mesenchymal transition. In addition, BAY1143269 suppresses eIF4E phosphorylation, inhibits cell proliferation, and induces apoptosis of glioblastoma cells. This drug also promoted a significant tumor growth reduction in a xenograft mice model (Wan et al., 2022).

Another drugs act on the MNK1/2-eIF4E axis with the outcome of eIF4E Ser209 dephosphorylation (Figure 7A). Some of them are currently being evaluated in clinical trials for their capacity to reduce tumor size or cell proliferation (Abdelaziz et al., 2021; Xu et al., 2022). Chen et al. (2021) studied the antitumor activity of Osimertinib, an FDA-approved EGFR inhibitor against primary GB cells isolated from patients. They found that this drug inactivated MNK1/2 and also led to eIF4E dephosphorylation. Moreover, in GB patient-derived xenotransplants in mice, oral administration of Osimertinib suppressed tumor growth and inhibited eIF4E phosphorylation in tumor cells. CGP57380 and Cercosporamide are drugs found to be able to inhibit eIF4E and GB growth in mice (Grzmil et al., 2014). In another study, Bell et al. (2016) demonstrated that pharmacological MNK inhibition with LY2801653 (Merestinib) targeted mesenchymal glioma stem cells and was able to prolong survival in a mouse model of GB.

Combinations of chemical agents are also being tested to treat GB. For example, a combination of compounds targeting both MNK and mTOR was reported to significantly sensitize GB cells to the mTOR inhibitor rapamycin resulting in a reduction of cell proliferation (Hemmings et al., 2010). In GB cells, the MNK1 inhibitor CGP-57380 inhibited eIF4E phosphorylation, and siRNA-mediated knockdown of MNK1 expression reduced the proliferation of cells incubated with rapamycin and CGP-57380 (Figure 7A). Concomitant with eIF4E dephosphorylation, this compound downregulated cell proliferation and colony formation (Grzmil et al., 2011). Cercosporamide, in combination with rapamycin analogs (rapalogs), significantly blocked GB tumor growth in mice (Grzmil et al., 2014). Other studies have shown that TMZ promotes eIF4E phosphorylation by MNK1/2 in glioma cells. Accordingly, inhibition of MNK1/2 activity using drugs or protein knockdown sensitized GB cells to TMZ treatments, which prevented cell growth (Bell et al., 2016; Grzmil et al., 2016; Xu et al., 2021). Finally, ribavirin alone or in combination with TMZ and interferon-β (IFN-β) inhibited growth, impaired glioma cell adhesion, and migration of GSCs glioma cells by inducing cell cycle arrest in the G0/G1 phase (Ogino et al., 2014; Volpin et al., 2017; Ochiai et al., 2020). *In vivo* experiments showed that ribavirin significantly extended the median survival and maximized the cytotoxicity effects of TMZ and radiation, increasing cell death in glioma and GSC (Volpin et al., 2017) (Figure 7A).

### Targeting the PI3K/AKT/mTOR cascade that controls eIF4E activity

In response to environmental stimuli and cellular cues, the PI3K/AKT/mTOR cascade controls cell growth, survival, and proliferation *via* the regulation of lipids, nucleotide, and protein synthesis. As described, dephosphorylated 4E-BPs trigger eIF4E/4E-BP complex formation and inhibit eIF4E/eIF4G interaction, thereby repressing cap-dependent translation. The PI3K/AKT/mTOR pathway is frequently dysregulated in different cancer types with consequences in cell proliferation, survival, migration and invasion (Fonseca et al., 2016; Roux and Topisirovic, 2018). The involvement of this pathway in GB is well established. mTOR signaling is hyperactivated in GB, which promotes protein synthesis and tumor size (Rich et al., 2005; Holand et al., 2011; Gini et al., 2013; Langhans et al., 2017; Mecca et al., 2018). Indeed, phosphorylated 4E-BP (p-4E-BP) levels significantly correlated with tumor grade and patient survival (Korkolopoulou et al., 2012).

Inhibiting the PI3K/AKT/mTOR signaling has emerged as a strategy to treat GB, and different small molecule inhibitors, alone or in combination, have shown promising results (Figure 7B) (Behrooz et al., 2022; Liu et al., 2022). Chlorpromazine inhibited the PI3K/AKT/mTOR cascade, arrested cell cycle in the G2/M phase, and promoted autophagy-induced cell death in GB cells (Shin et al., 2010; Tanaka et al., 2015; Oliva et al., 2017). The anti-depressant Fluvoxamine acts on this signaling cascade too, and decreases cell invasion, migration, and lamellipodia formation in GB cells. Fluvoxamine also prolonged a mice model’s survival (Hayashi et al., 2016). On the other side, Imipramine strongly reduced colony formation and mitochondrial activity in U87MG and C6 GM cells (Jeon et al., 2011; Shchors et al., 2015).

Currently, more than 50 PI3K or dual PI3K/mTOR inhibitors are being developed and tested in cancer therapies (Behrooz et al., 2022; Liu et al., 2022). However, only a few have shown promising results and have entered clinical trials. The more relevant include Buparlisib, which can reduce GB cell growth both *in vitro* and in tumors (Burger et al., 2011; Koul et al., 2012; Wen et al., 2019); BKM120 showed anti-tumor activity in GB cells and in mice BALB/c that were inoculated with U-87 MG cells (Fan Q. et al., 2017); Pilaralisib and Voxtalisib alone or in combination with conventional therapies containing TMZ have shown tumor suppression activities (Figure 7B) (Prasad et al., 2011; Wen et al., 2015; Gravina et al., 2016; Wheler et al., 2017); and Metformin, a drug used to treat type 2 diabetes, sensitized GB and GB stem cells to TMZ, a strategy that has entered early clinical trials (Yu et al., 2015; Byron et al., 2018).

### Targeting eIF4A

Silvestrol is an organic heterocyclic compound isolated from the tropical plant *Aglaia silvestris* that exhibits anticancer activity. Silvestrol is an eIF4A inhibitor able to kill tumor cells in colorectal carcinoma, acute lymphoblastic leukemia, breast, and prostate cancer in mice and human cells (Figure 7C) (Cencic et al., 2009; Alachkar et al., 2013; Kogure et al., 2013). Interestingly, Silvestrol exerts antitumor effects in U251 and U-87 MG GB cells by inhibiting the expression of Cyclin D1, the PI3K/AKT/mTOR, and ERK1/2 signaling cascades (Zhang et al., 2022) (Figure 7C). Currently, the molecular mechanism underlying these observations are not well understood.

### Targeting mRNA IRES

Internal ribosome entry site (IRES)-dependent mRNA translation is a cap-and eIF4E-independent mechanism that places the 43S PIC at or very near the AUG TIS without the involvement of eIF4E. IRES-dependent translation is involved in tumor growth, survival, and chemoresistance in GB cell lines (Holmes et al., 2016). The *c-Myc* oncogene and Cyclin D1 mRNA translation is driven by IRESs and the IRES trans-acting factors (ITAFs) hnRNP A1 (heterogeneous nuclear ribonucleoprotein A1) (Shi et al., 2005; Martin et al., 2011). Holmes identified a class of inhibitors called C11 and IRES-J007, which block the ability of the ITAF hnRNP A1 from associating with Cyclin D1 and c-Myc IRES, leading to the downregulation of mRNA translation (Figure 7D) (Holmes et al., 2016). The same research group subsequently identified Riluzole, an FDA-approved drug for the treatment of amyotrophic lateral sclerosis, that markedly blocked both Cyclin D1 and c-Myc IRES activity. Riluzole interacts with identical residues as C11 and IRES-J007 and His120 on hnRNP A1 *via* hydrogen bonding (Benavides-Serrato et al., 2020). *In vivo* co-therapy with Riluzole and the mTORC1/2 inhibitor PP242 in xenografted mice demonstrated significant tumor growth inhibition, increased apoptotic cell death, and the overall survival of mice significantly extended (Benavides-Serrato et al., 2020). Moreover, a Riluzole/TMZ co-therapy demonstrated the strongest synergism and inhibitory effect on cell proliferation in GB MGMT-positive cell lines and suppressed tumor growth *in vivo*. Interestingly, Riluzole in combination with TMZ therapy, avoided the TMZ-induced upregulation of MGMT through the inhibition of MGMT mRNA expression in GB (Yamada et al., 2020).

### Targeting the mitoribosome

Increasing evidence shows that targeting the mitochondrial oxidative phosphorylation (OXPHOS), electron transport chain activity, and mitochondrial translation could be clinically exploitable in GB therapies for TMZ-resistant glioma cells and GSCs, which are responsible for TMZ therapy failure and disease recurrence (Oliva et al., 2010; Kuramoto et al., 2020). FDA-approved combination of the antibiotics Quinupristin and Dalfopristin (Q/D), used to treat bacterial infections, could be used to treat GB (Figure 7E) (Manzella et al., 2001). Sighel et al. proved that Q/D promote significant growth inhibition of GSCs. These molecules bind to the large mitoribosome subunit (dalfopristin at the peptidyl-transferase center and quinupristin at the begining to the exit tunnel for nascent polypeptides), stalling polypeptide synthesis, which dysregulates the activity of OXPHOS complexes I, IV, and V (Sighel et al., 2021). This dysregulation led to loss of clonogenic potential, cell cycle arrest, and death by apoptosis in GSCs in normoxia and hypoxia (Sighel et al., 2021).

## Outlook

Malfunctioning translational control and the translation machinery are emerging as causes of GB onset and development. However, to our knowledge, no translation elongation or release factors have been reported to play a role in GB progression, which remains an unexplored field. Some chemical compounds targeting translation initiation factors and the cascades signaling to translation initiation are being tested to fight GB at the experimental level and soon will go through clinical assays. So far, no chemical compound has been tested to target RP for GB treatment. Investigating elongation and release factors in GB and the develop of novel drugs to target RP could give rise to new and exciting fields.

Interestingly, in different types of tumors other than GB, a myriad of pharmacological compounds is being assessed in preclinical or clinical trials to target translation factors and the MNK1/2-eIF4E axis. These include dihydrotestosterone (Overcash et al., 2013), Salubrinal (Boyce et al., 2005), and BTdCPU (Chen et al., 2011) to inhibit eIF2; Hippuristanol (Bordeleau et al., 2006), Pateamine (Hood et al., 2001), and Flavagline (Basmadjian et al., 2013) to inhibit eIF4A; 4E1RCat (Cencic et al., 2011), 4Ei-1 (Ghosh et al., 2009), and 4EASO (Jacobson et al., 2013) to inhibit eIF4E; 4EGI-1 (Takrouri et al., 2014) to block the eIF4E/eIF4G association; and QL-X-138, Pyrrolopyrimidine-derivative compounds, eFT508, and ETC-206 to inhibit MNK1/2 and eIF4E phosphorylation (Konicek et al., 2011; Diab et al., 2016; Wu et al., 2016; Winter-Holt et al., 2017; Teneggi et al., 2020; Conn et al., 2021). Thus, exploring this broad spectrum of drugs alone or in various combinations to treat GB could elicit unsuspected and promising results.

We expect that further studies on the involvement of the translation machinery in GB and the development of new drugs to treat it will soon improve the survival outcome of patients and take GB out of the spotlight. Hopefully, soon, the dark side of translation in GB will become clear.
